# Protective role of fingolimod (FTY720) in rats subjected to subarachnoid hemorrhage

**DOI:** 10.1186/s12974-015-0234-7

**Published:** 2015-01-27

**Authors:** Hao-Liang Xu, Dale A Pelligrino, Chanannait Paisansathan, Fernando D Testai

**Affiliations:** Neuroanesthesia Research Laboratory, University of Illinois College of Medicine, Chicago, IL USA; Department of Anesthesiology of the University of Illinois College of Medicine, Chicago, IL USA; Department of Neurology and Rehabilitation of the University of Illinois College of Medicine, 912 S Wood Street, Rm 855 N NPI (MC 796), Chicago, IL 60612-7330 USA

**Keywords:** Subarachnoid hemorrhage, Microvascular function, Fingolimod, Neuroinflammation, Cerebrovascular disease/stroke, Subarachnoid hemorrhage

## Abstract

**Background:**

Subarachnoid hemorrhage (SAH) is a neurological emergency with limited pharmacological treatment options. Inflammation is increasingly recognized as a key pathogenic contributor to brain injury in this condition. In the present study, we examined the neuroprotective effects of the immunomodulatory agent, fingolimod, in rats subjected to SAH.

**Methods:**

We utilized an endovascular rat perforation model of SAH. Animals were divided into four groups: (1) sham-vehicle; (2) sham-fingolimod; (3) SAH-vehicle; and (4) SAH-fingolimod. Rats received either vehicle solution or fingolimod (0.5 mg/kg) intraperitoneally 3 hours after sham surgery or SAH. A closed cranial window and intravital microscope system was used at 48 hours to assess neuroinflammation, which was represented by rhodamine-6G-labeled leukocyte trafficking in pial venules, and pial arteriolar dilating responses to a variety of vasodilators, including hypercapnia, and topically-applied acetylcholine, adenosine, and S-nitroso-N-acetyl penicillamine. In addition, motor-sensory function was evaluated.

**Results:**

Compared to sham-vehicle rats, SAH-vehicle animals displayed a four-times greater increase in pial venular intraluminal leukocyte adhesion. Treatment with fingolimod largely reduced the intravascular leukocyte adhesion. Vehicle-treated SAH animals displayed a significant decrease in pial arteriolar responses to all the vasodilators tested and vascular reactivity was preserved, to a significant degree, in the presence of fingolimod. In addition, neurological scores obtained at 48 hours post-SAH indicated significant neurological deficits in the vehicle-treated group (versus sham-vehicle surgical control). Those deficiencies were partially reduced by fingolimod (*P* < 0.0001 compared to the vehicle-treated SAH group).

**Conclusions:**

Treatment of rats with fingolimod was associated with a marked limitation in the intravascular adhesion of leukocytes to pial venules, preserved pial arteriolar dilating function, and improved neurological outcome in rats subjected to SAH.

## Introduction

Subarachnoid hemorrhage (SAH) is associated with a disproportionately elevated morbidity and mortality. The overall case fatality is estimated in 40 to 50% with almost 10 to 15% of the patients dying before reaching medical attention [1,2]. Of the survivors, 46% may have long-term cognitive impairment and 30% require lifelong supportive care [3,4]. This condition often affects young adults and may be responsible for almost a quarter of all the years lost because of stroke [5]. Despite intense research, progress toward the development of effective treatments for SAH has been disappointingly slow. Nimodipine remains the only pharmacological treatment for this condition. The beneficial effect of nimodipine, however, is limited and its use is often restricted by the occurrence of hypotension [6]. New and effective treatments for SAH are largely needed. Studies performed over the past decade have yielded a preponderance of evidence supporting a link between inflammation and stroke outcome [7-10]. Inflammation plays an important role in brain damage by contributing to neural injury, diminished vascular reactivity, microthrombosis, and enhanced blood brain barrier (BBB) permeability [7,8,11,12]. These observations support immunomodulation as a potential target for intervention following SAH. Fingolimod (FTY720) is an immunomodulatory agent has been approved by the Food and Drugs Administration (FDA) for the treatment of multiple sclerosis. Fingolimod is a sphingosine-1-phosphate (S1P) analog that crosses the BBB and regulates critical cellular processes including proliferation, apoptosis, endothelial barrier permeability, and inflammation [13,14]. Studies performed in association with cerebral ischemia and intraparenchymal hemorrhage have shown that FTY720 reduces stroke-related neuroinflammation, brain edema, and neuronal death and improves neurological outcome [15-17]. Thus, we hypothesized that fingolimod would be efficacious in the treatment of SAH. To that end, we investigated the effect of FTY720 in a rat model of SAH. In particular we addressed leukocyte adhesion to pial venules, microvascular (pial arteriolar) dilating reactivity, and neurological outcome.

## Methods

### Drugs and SAH model

FTY720 was supplied by Cayman Chemicals (Ann Arbor, MI, USA). Experimental protocols were approved by the institutional Animal Care and Use Committee of the University of Illinois at Chicago. Sprague–Dawley male rats (250 to 300 g) were randomly assigned to the sham-vehicle (n = 10), sham-FTY720 (n = 10), SAH-vehicle (n = 11), or SAH-FTY720 (n = 11) groups. We utilized the endovascular perforation model of SAH as previously described [18]. Briefly, animals were anesthetized with 2% isoflurane and mechanically ventilated. Physiological variables, including blood pressure, blood gases, and body temperature, were continuously monitored and kept within physiological ranges. Regional cerebral blood flow (rCBF) was monitored before, during, and after SAH by Laser Doppler flowmetry (LDF), which entailed attachment of a laser-Doppler probe to the skull over the right middle cerebral artery (MCA) territory. The right internal (ICA) and external carotid arteries (ECA) were isolated to their origins at the common carotid artery (CCA) bifurcation. The ECA was ligated and shaped into a short stump. The CCA was temporary clipped, and a hollow polytetrafluoroethylene (PTFE) tube was advanced rostrally into the ICA from the ECA stump until resistance was felt, and then a tungsten wire was partially advanced through PTFE tube to perforate the bifurcation of the anterior and middle cerebral arteries. Immediately after puncture, the PTFE tube and tungsten wire were retracted into the ECA stump, and the ICA was reperfused. The incision was then closed with nylon monofilament suture, and rats were extubated and returned to their cages. SAH was determined by a transient drop in cerebral blood flow of > 95% and confirmed by post-mortem examination which was performed at 48 hours post-surgery. Blood accumulation at the circle of Willis indicates a successful vessel perforation. Similar surgical procedure was performed in sham-vehicle and FTY720 groups except that the suture was removed once resistance was felt and no tungsten wire perforation was performed. To minimize factors that may influence bioavailability we used the intraperitoneal (IP) route. Animals were treated with vehicle or FTY720 (0.5 mg/kg) at 3 hours after surgery.

### Peripheral leukocyte count

The effect of FTY720 on leukocytes was determined using peripheral blood samples. Animals were randomly assigned to sham-vehicle (n = 3), sham-FTY720 (n = 5), SAH-vehicle (n = 5), and SAH-FTY720 (n = 4) groups. Blood samples were collected before (baseline) and 48 hours after surgery. An additional non-surgical naïve control group was used to confirm the immonomodulatory effect of FTY720. A total of 0.5 ml of blood was obtained from the subclavian vein, placed in a Microtainer tube coated with K_2_-EDTA (BD Biosciences, San Jose, CA, USA), and analyzed using an automated hematology analyzer Advia 120 (Siemens, Dublin, Ireland).

### Cranial window installation

Cranial windows were installed 48h after surgery as previously described. Briefly, bilateral femoral arterial and venous catheters were placed with the rats under 2% isoflurane/70%N_2_O/30%O_2_ anesthesia, paralyzed with curare (1 mg/kg intravenously) and supported with mechanical ventilation. Femoral artery and vein were canulated for arterial blood pressure monitoring and arterial blood gas measurement. Body temperature was kept at 37°C. The animal was positioned on a stereotactic frame and a 10 mm craniotomy was performed over the skull midline. The dura was removed carefully to keep the sagital sinus intact. An 11 mm in diameter and 1.5 mm in thickness borosilicate glass window equipped with 3 ports was glued to the skull. The time from dural excision to sealing of the cranial window was minimized (less than 5 minutes) to avoid complications from brain swelling and damage at the dural edge. The intracranial pressure (ICP) under the cranial window was monitored thorough the experiment using the ICP measurement port. Artificial CSF (aCSF) was suffused into the cranial window and the effusion rate was adjusted to maintain an ICP of 7 to 11 mmHg in all groups. After completion of the surgery, the anesthesia with isoflurane was switched to fentanyl infusion for monitoring of rhodamine-6G (R6G) leukocyte labeling and the assessments of pial arteriolar responses.

### Leukocyte behavior analysis

Leukocyte behavior analyses were performed 48 hours after surgery by infusing R6G as previously described [19]. This is a semiquantitative method used for the *in vivo* assessment of leukocyte trafficking. Fluorescence area of R6G-labeled leukocytes adhered to the vessel wall (this includes both tightly-adhered and slow-rolling leukocytes) is used as a surrogate marker of neuroinflammation. Animals were anesthetized with 70% N_2_O/30% O_2_ - fentanyl (25 μg/kg/h, intravenously). After cranial window placement, R6G (0.2 mg loading dose) was applied intravenously to sham-vehicle, sham-FTY720, SAH-vehicle, and SAH-FTY720 groups, followed by 0.2 mg/h maintenance dose (n = 6 per group). Pial venules ranging from 40 to 80 μm in diameter were randomly selected. R6G-labeled stationary leukocytes were viewed and captured using a digital camera (CoolSnap ES, Photometrics, Tucson, AZ, USA) mounted on a fluorescence microscope (Nikon, Melville, NY, USA) equipped with an epi-illumination dark field system. Images were obtained 60 minutes after initiating R6G infusion and analyzed using MetaMorph software (Molecular Devices, Downingtown, PA, USA). R6G dosage, intravenous suffusion time, and laser light source were applied in identical manner to each animal, minimizing the variations in leukocyte labeling. Using this technique, only adherent leukocytes can be detected [20]. Leukocyte adhesion was reported as the % of adherent leukocytes occupying the viewed venular area in the microscopic field.

### Arteriolar reactivity

Vascular reactivity was determined 48 hours after SAH by measuring pial arteriolar diameter changes in response to different vasodilating stimuli, including hypercapnia, acetylcholine (Ach, 10 μM and 100 μM), adenosine (ADO, 10 μM and 100 μM), and S-nitroso-N-acetyl penicillamine (SNAP, 0.1 μM and 1 μM) as previously described [19,21]. Briefly, animals were randomly assigned to the sham-vehicle, sham-FTY720, SAH-vehicle, and SAH-FTY720 groups (n = 5 to 6 per group). Pial arteriolar diameters were measured through a cranial window after 40 minutes of suffusion of aCSF (baseline) and again after 3 minutes of hypercapnia (PaCO_2_ at approximately 70 mmHg). CO_2_ reactivity was calculated as the percentage of diameter increase per mmHg of PaCO_2_ change. Fifteen minutes after return to baseline, Ach, ADO, and SNAP were sequentially suffused in the space under the cranial window at the concentrations indicated. Diameter values from three arteriolar segments were obtained and averaged.

### Neurological assessments

These were done at 48 hours post-SAH by a blinded observer prior the placement of the cranial window. We utilized a 21-point scale as previously described [20]. Briefly, animals were randomly assigned to the sham-vehicle, sham-FTY720, SAH-vehicle, and SAH-FTY720 groups (n = 10 to 11 per group). The ratings were taken from a sum of scores from 7 categories: (i) spontaneous activity, (normal = 3, akinesia = 0); (ii) side stroking (bilateral brisk response = 3, no response = 0); (iii) vibrissae touch (bilateral brisk response = 3, no response = 0); (iv) limb symmetry (forelimb and hindlimb extended = 3, contralateral forelimb and hindlimb completely flexed = 0); (v) lateral turning (bilateral turning ≥ 45° = 3, no turning = 0); (vi) forelimb walking (briskly walks forward in symmetry = 3, cannot move on forelimbs = 0); and (vii) slope climbing (climbs to the top of slope with prompting and strong grip = 3, weak grips and falls off = 0).

### Statistics

Group sizes were determined based on power analysis. In our calculations we assumed a 30-50% change in the mean, 20% standard deviation (SD), alpha of 0.05, and power of 0.8. Based on power analysis the minimum group sizes were 5. Peripheral leukocyte counts were expressed as means ± SD and analyzed using an unpaired Student *t-*test. Venular leukocyte adhesion, pial arteriolar reactivity, and neurological outcome were expressed as means ± standard error of the mean (SEM). The effect of FTY720 on on these variables was analyzed using one-way analysis of variance (ANOVA). Significance was defined as *P* < 0.05.

## Results

### Physiological variables for the SAH animal model

**Figure 1:**
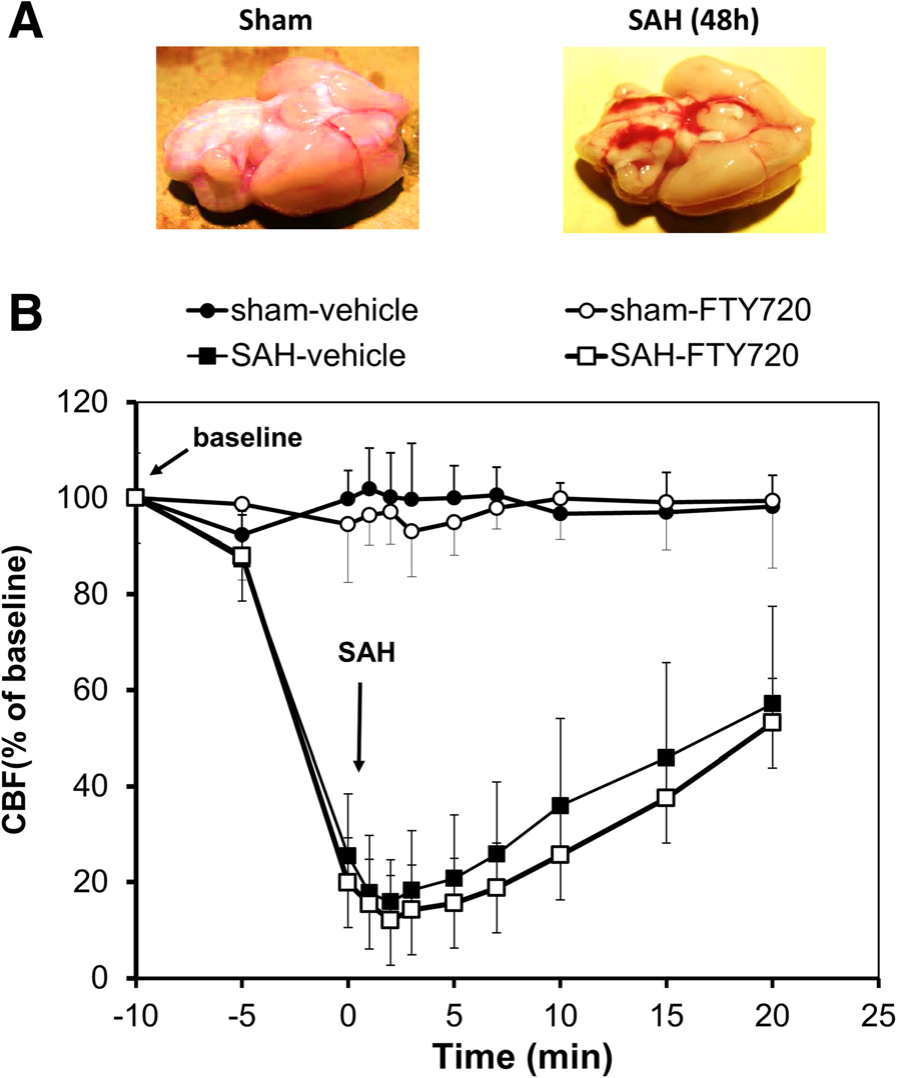
**Experimental model. (A)** Representative post-mortem examinations of brains isolated from vehicle-sham controls and vehicle-SAH animals. **(B)** Cerebral blood flow (CBF) measured in the sham-vehicle (closed circles), sham-FTY720 (open circles), SAH-vehicle (closed squares), and SAH-FTY720 (opened squares) groups. Time zero represents the time of surgery. The SAH-FTY720 group was treated 3 hours after surgery. n = 10 to 11 per group. SAH, subarachnoid hemorrhage.

The overall mortality after SAH was approximately 30%. Of the animals that survived the SAH surgery, one animal died in the SAH-vehicle group and one in the SAH-FTY720 group. Blood pressure, body temperature, and blood gas parameters were similar in the four groups. CBF decreased abruptly by 85 to 90% after SAH. The nadir occurred within the first minute of SAH and this was followed by a gradual recovery reaching a plateau at approximately 60% of baseline values within 20 minutes. The cerebral hemodynamic responses were comparable in both the vehicle- and FTY720-groups. SAH was also confirmed by post -mortem visual inspection (Figure 1).

### Effect of FTY720 on circulating blood cell counts

**Figure 2:**
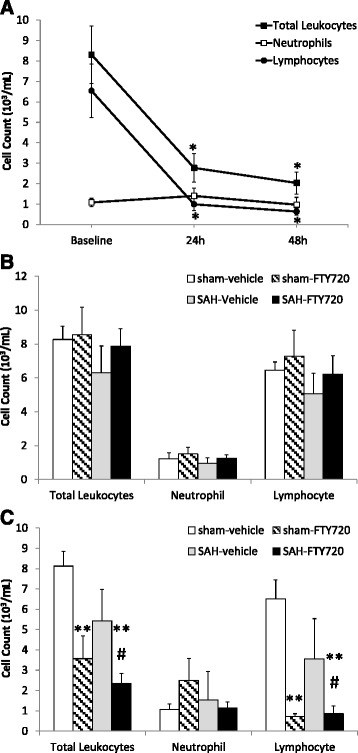
**Peripheral leukocyte cell count.** Non-surgical animals (n = 3) received a single dose of fingolimod (0.5 mg/kg) and peripheral blood obtained at baseline, 24 hours and 48 hours post-treatment **(A)**. Peripheral venous blood was also obtained from sham-vehicle, sham-FTY720, SAH- vehicle and SAH-FTY720 animals at baseline **(B)** and 2 days after surgery **(C)** (n = 5 to 6 per group). Leukocyte cell counts were determined as indicated in Methods. Statistical significance was determined by Student *t*-test. Bars represent means ± SD. *P < 0.001 versus baseline. **P < 0.001 versus vehicle-sham. # *P* < 0.05 versus Vehicle-SAH. SAH, subarachnoid hemorrhage.

The treatment with a single dose of 0.5 mg/kg FTY720 in non-surgical animals resulted in a steep decrease in circulating white blood cells at both 24 hours and 48 hours (Figure 2A). This decrease was largely driven by lymphocyte depletion, which decreased to 15% and 12% from the basal levels measured at 24 hours and 48 hours post-treatment, respectively (*P* < 0.001). Blood samples in sham and SAH-exposed animals were obtained at baseline and 48 hours after surgery. In comparison to vehicle-SAH animals, a single dose of 0.5 mg/kg FTY720 3 hours after sham- or SAH-surgery was associated with a statistically significant decrease in total leukocytes and lymphocytes in peripheral blood (Figure 2B and C).

### Leukocyte adhesion

**Figure 3:**
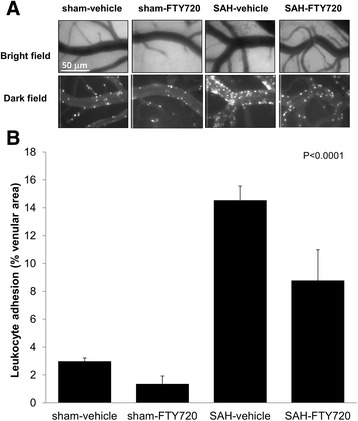
**Leukocyte vascular adhesion.** Animals were treated with vehicle or FTY720 (0.5 mg/kg) 3 hours post-surgery and leukocyte adhesion was determined at 48 hours. **(A)** Representative pictures of the vessel anatomy (bright field) and the trafficking of leukocytes (dark field) 1 hour after leukocyte labeling with R6G in sham-vehicle, sham-FTY720, SAH-vehicle, and SAH-FTY720-treated animals. **(B)** Quantification of leukocyte adhesion, expressed as the percentage of the vessel area occupied by adherent leukocytes measured 1 hour after leukocyte labeling. Bars represent means ± SEM. n = 6 each group. Significance was determined using ANOVA. SAH, subarachnoid hemorrhage.

Circulating leukocytes were tagged with R6G and video-recorded at 48 hours post-SAH. Representative frames were captured from video-recordings (Figure 3). Leukocyte-adhesion in the sham-vehicle and sham-FTY720 groups were 2.9 ± 0.2% and 1.3 ± 0.5%, respectively. Vehicle-SAH animals displayed almost an approximately 5-fold increase in vascular leukocyte adhesion to pial venules compared to controls (14.4 ± 1.0%; *P* < 0.0001). Treatment with FTY720, on the other hand, was associated with a decreased SAH-induced leukocyte adhesion by approximately 40% (8.8 ± 2.2%).

### Arteriolar reactivity

**Figure 4:**
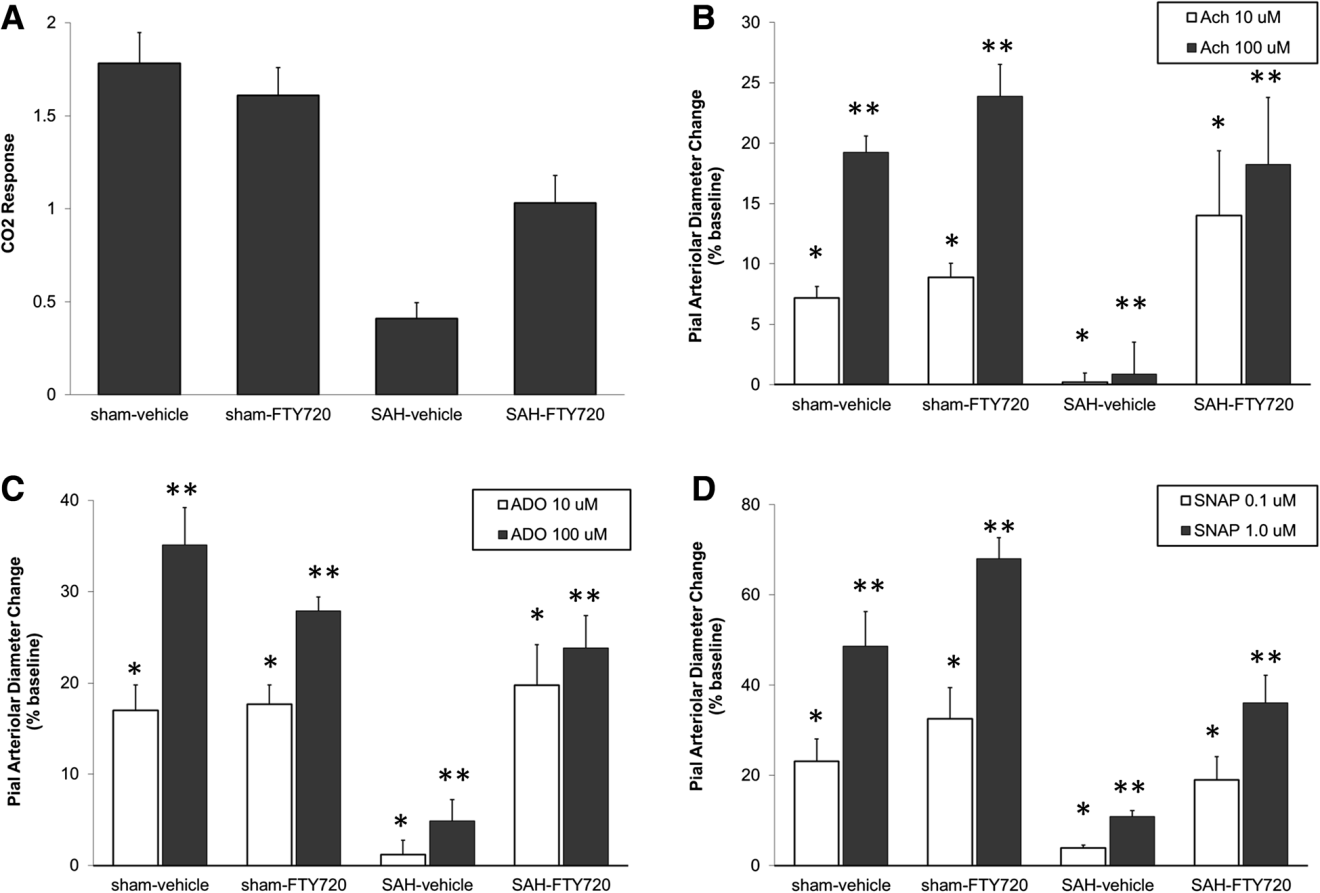
**Pial arteriolar dilating reponses.** Animals were treated with vehicle or FTY720 (0.5 mg/kg) 3 hours post-surgery. Measurements were done at 48 hours after. Compared to SAH-vehicle, SAH-FTY720 animals had improved pial arteriolar vasodilating responses to all the vasodilators tested. **(A)** hypercapnia (PaCO_2_ at approximately 70 mmHg) (*P* < 0.001). **(B)** acetylcholine, Ach (*P = 0.003; **P = 0.002). **(C)** adenosine, ADO (*P = 0.002; **P < 0.001). **(D)** S-nitroso-N-acetyl penicillamine, SNAP (*P = 0.007; **P < 0.001). Bars represent means ± SEM. n = 5 to 6 each group. Significance was determined using ANOVA.

Cerebral microvascular dilating function was determined by assessing arteriolar reactivity to established vasodilators, including hypercapnia (PaCO_2_ at approximately 70 mmHg), Ach (10 μM and 100 μM), ADO (10 μM and 100 μM), and SNAP (0.1 μM and 1 μM). Time-dependent alterations in arteriolar reactivity occur in SAH [22]. These changes reach their maximum 48 hours and gradually recover in the following 7 days after the hemorrhage. Hence, arteriolar reactivity was determined at 48 hours post-SAH. The arteriolar responses in the sham-vehicle and sham-FTY720 were comparable. SAH-vehicle animals showed a profound attenuation of pial arteriolar responses to all the vasodilators tested. In comparison, SAH-FTY720 animals displayed a statistically significant preservation of arteriolar dilating function to all the agents investigated (Figure 4). The improvement was partial for hypercapnia and virtually complete for Ach, ADO, and SNAP.

### Neurological outcome

**Figure 5:**
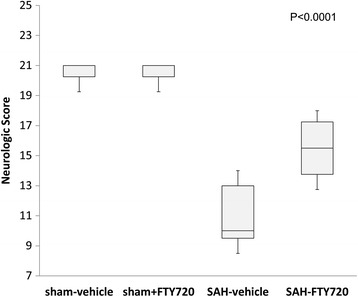
**Neurological score.** Animals were treated with vehicle or FTY720 (0.5 mg/kg) 3 hours post-SAH and the neurological score determined at 48 hours after SAH. N = 10 to 11 each group. Significance was determined using ANOVA.

Functional outcome was determined at 48 hours post-SAH (Figure 5). The neurological score in sham-vehicle and sham-FTY720 groups were not statistically different (20.5 ± 0.4 versus 20.6 ± 0.4). In comparison, the score was reduced to 11.4 ± 2.4 in the SAH-vehicle group. The treatment with FTY720 at 3 hours after SAH was associated with an improvement of 35% in neurological outcome (15.5 ± 1.8; *P* < 0.0001).

## Discussion

In this study we demonstrated that the use of fingolimod was associated with a restricted leukocyte adhesion to pial vessels, preserved arteriolar vasodilation responses, and improved neurological function after SAH.

FTY720 has been used with success in models of cerebral ischemia and parenchymal hemorrhage [15-17]. This, however, is the first study addressing the protective effect of this drug in animals subjected to SAH. The dose of FTY720 is in agreement with studies done in non-SAH models [15,17,23,24]. SAH is characterized by acute headache resulting in patients seeking early medical attention. Therefore, the 3 hours post-SAH treatment time point constitutes a therapeutic window similar to that encountered in clinical practice.

FTY720 is an agonist of S1P receptors type 1, 3, 4 and 5 which are ubiquitously distributed in cells of different lineages. Fingolimod induces the internalization and subsequent ubiquitination and proteosomal degradation of S1P-receptor type 1 on lymphocytes, which are retained in lymph organs [25]. In our study, FTY720 decreased circulating lymphocytes with virtually no effect on neutrophils, an observation that is consistent with the known mechanism of action of this drug [25,26]. FTY720 has a high volume of distribution and its half-life is of approximately 9 to 10 days [14]. Thus, it was not unexpected that a single dose of this drug led to an immunosuppressive state that persisted for at least 48 hours. In addition, the treatment with FTY720 restricted the adhesion of leukocytes to pial vessels and improved neurological outcome after SAH. The role of leukocytes in SAH is not well known. Immediately after SAH, there is a rapid increase in the ICP leading to transient global hypoperfusion with subsequent restoration of the cerebral blood flow [27]. In this context, SAH has some similarities with transient cerebral ischemia. Studies performed in ischemic models illustrate that tissue hypoxia upregulates the expression of adhesion molecules that facilitate the interaction of circulating leukocytes with vascular elements. These release vasoactive and pro-inflammatory mediators that contribute to brain damage [10,28,29]. In SAH, the initial insult induces an inflammatory state characterized by early recruitment of leukocytes and increased production of cytokines and chemokines in both plasma and CSF [9,12,30]. This immune response correlates with SAH severity and has been linked to neural cell injury, BBB dysfunction and delayed cerebral ischemia [31-33]. Accordingly, immunomodulation has emerged as a potential treatment strategy that could ameliorate brain injury and improve outcome after SAH. Our previous findings indicate that blocking leukocyte adhesion and transmigration into the brain parenchyma via LJP-1586, a selective vascular adhesion protein-1 (VAP-1) inhibitor, improves neurological outcome in SAH [34]. LJP-1586 is nonspecific and inhibits the adhesion of neutrophils, monocytes and lymphocytes. In comparison, FTY720 has a selective effect on lymphocytes. Studies performed in ischemic models confirm the contribution of both innate and adaptive immunity to stroke outcome. Understanding the role of lymphocytes in cerebral ischemia is confounded by the existence of specific subpopulations that are either protective, deleterious, or both [29]. Despite the limited information regarding the specific contribution of lymphocytes to SAH outcome, the selective effect of FTY720 supports the key role of this cell type on SAH-associated brain injury. Animal models, however, do not necessarily recapitulate the complex pathogenic mechanisms that take place in humans. From the immunological standpoint, the predominant circulating leukocytes in humans are neutrophils (50 to 70%) and in rodents lymphocytes (60 to 80%) [35]. In addition, immune-related molecules are differentially expressed in different species [35]. Therefore, the results obtained in experimental models may not necessarily apply to humans. Both similarities and differences have been described in the immune responses triggered by SAH in rodents and humans [31]. Significantly, it was observed that methylprednisolone, a drug that preferentially targets lymphocytes, is beneficial in rodents and patients with SAH [36-38]. The studies were too small to draw solid conclusions but they support the active role of lymphocytes in SAH-associated brain injury.

In our study we also observed that the use of FTY720 preserved microvascular dilating function which was tested using different vasodilating agents. Hypercapnia and Ach induce cerebral arteriolar dilation via nitric oxide (NO) synthase/cyclooxygenase and endothelium-dependent mechanisms, respectively. In comparison, ADO tests G-coupled receptor-dependent vascular smooth muscle responses and SNAP interrogates vascular smooth muscle NO reactivity. Time-dependent alterations in the response of small arteries to vasodilators occur within minutes of the SAH and peak at 48 hours [22]. The upstream processes governing vascular tone on SAH are not completely understood. An association between inflammation and abnormal vasomotor responses in SAH has been established in large conduit arteries [39,40]. Lymphocytes, in particular, have been associated with the emergence of late vasospasm [41]. Pharmacological blockage of VAP-1 and neutrophil inhibition, in addition, ameliorate microvascular dysfunction in animal models suggesting that immunological factors regulate vasoreactivity in both small and large intracranial vessels [39]. Interestingly, there is evidence supporting the notion that microvascular dysfunction is a major contributor to microthrombi formation, tissue hypoxia, and outcome after SAH [42-44]. The improvement in neurological outcome, in parallel with decreased neuroinflammation and preserved arteriolar responses observed in association with FTY720, is in agreement with this paradigm. It should be pointed out, however, that this drug has a direct stabilizing effect on the endothelium and has been shown to enhance the endothelial synthesis of NO in non-neural tissues [14,45]. In our study the arteriolar responses in the sham-vehicle and sham-FTY720 were comparable (*P* > 0.05) indicating that, under the conditions used in this study, FTY720 does not have a direct vasodilating effect. FTY720 has a direct epigenetic effect via histone deacetylases regulation and also targets different components of the neurovascular unit. In particular, it inhibits astrogliosis, increases neuronal survival in cerebral ischemia, and enhances endothelial-barrier function [25,46-48]. Therefore, the preserved arteriolar function and neurological improvement observed in our study cannot be conclusively ascribed to the inhibition of lymphocytes.

Studies done in ischemia models demonstrate that FTY720 decreases brain edema, reduces neuronal cell death, and improves long-term neurobehavioral outcome [15,17,49] Furthermore, the early treatment of intracerebral hemorrhage patients with FTY720 reduces hematoma-associated edema and improves neurological outcome at 3 months [50]. Since SAH is associated with unique pathogenic mechanisms, the extrapolation of findings gathered in other stroke models should be done with caution. Current data, however, demonstrate that FTY720 has pleiotropic effects and can influence key elements of SAH, including neuronal survival, BBB permeability, neuroinflammation, and microvascular dysfunction [15,17,49].

Similar to other brain-injury models, a profound immunodepression develops in SAH patients [51]. This temporary state lasts for at least 3 days after the bleed and is associated with a high rate of infections, particularly pneumonia. Thus, the potential translation of our findings to clinical practice may be limited by the immunomodulatory nature of fingolimod. Fingolimod is known to retain naïve T cells and central memory T cells in lymph nodes. However, this drug has a partial effect on peripheral effector memory T cells, which are important against infections [14]. There is limited information regarding the effect of fingolimod on infection rates on stroke. However, studies done in the rodent model of cerebral ischemia have shown that treatment with fingolimod reduces circulating leukocytes and improves outcome without increasing the rate of bacterial lung infections [52]. In addition, two recent small studies investigating the effect of fingolimod on patients with ischemic stroke and intracerebral hemorrhage showed similar rates of infection in both the treatment and placebo groups [50,53].

## Conclusion

Early treatment with fingolimod after SAH reduces leukocyte adhesion to pial venules, preserves arteriolar dilation function, and improves neurological outcome. Further studies are needed to determine the long-term effect of fingolimod on SAH outcome.

## Abbreviations

Ach: Acetylcholine
ADO: Adenosine
ANOVA: Analysis of variance
BBB: Blood brain barrier
CCA: Common carotid artery
CSF: Cerebrospinal fluid
ECA: External carotid arteries
FDA: Food and drug administration
FTY720: Fingolimod
ICA: Internal carotid artery
ICP: Intracranial pressure
IP: Intraperitoneal
LDF: Laser doppler flowmetry
MCA: Middle cerebral artery
NO: Nitric oxide
PTFE: Polytetrafluoroethylene
R6G: Rhodamine-6G
rCBF: Regional cerebral blood flow
S1P: Sphingosine-1-phosphate
SAH: Subarachnoid hemorrhage
SNAP: S-nitroso-N-acetyl penicillamine
VAP-1: Vascular adhesion protein-1
